# A randomized, controlled trial evaluating the efficacy of an online intervention targeting vitamin D intake, knowledge and status among young adults

**DOI:** 10.1186/s12966-016-0443-1

**Published:** 2016-11-11

**Authors:** Samantha Goodman, Barbara Morrongiello, Kelly Meckling

**Affiliations:** 1 Department of Human Health and Nutritional Sciences, University of Guelph, 50 Stone Road East, Guelph, N1G2W1 ON Canada; 2 Department of Psychology, University of Guelph, 50 Stone Road East, Guelph, N1G 2 W1 ON Canada

**Keywords:** Nutrition, Vitamin D intake, Vitamin D status, Mobile app, Behaviour change, Intervention, Emerging adulthood

## Abstract

**Background:**

Vitamin D plays a key role in bone health. Consuming adequate vitamin D during young adulthood is important due to the development of peak bone mass; however, many Canadian young adults do not meet vitamin D recommendations. This study aimed to improve knowledge, perceptions, dietary intake and blood concentrations of vitamin D among a sample of young adults.

**Methods:**

Using a pre-post design, 90 Ontario adults (38 men, 52 women; 18–25 years), were randomly assigned to intervention or control groups. Participants completed a socio-demographic survey, pre-post food frequency questionnaire, and a vitamin D knowledge questionnaire (3 time-points). The intervention group watched a video, received online information and tracked intake of vitamin D using a mobile application for 12 weeks. A sub-sample of participants completed pre-post blood 25(OH)D_3_ tests. Univariate ANOVA tested pre-post between-group differences in vitamin D intake and status. Repeated-measures ANOVA tested between-group differences in vitamin D knowledge and perceptions across 3 time-points.

**Results:**

Mean vitamin D intake in the sample increased significantly from pre-test (M = 407, SD = 460 IU) to post-test (M = 619, SD = 655 IU), *t*(88) = 5.37, *p* < 0.001. Mean intake increased significantly more in the intervention than control group after controlling for gender and education, *F*(1, 85) = 4.09, *p* = 0.046. Mean blood vitamin D_3_ was significantly higher among non-Caucasian than Caucasian participants at baseline, *t*(56.7) = 3.49, *p* = 0.001. Mean blood vitamin D_3_ increased significantly from pre-test (M = 28, SD = 16 nmol/L) to post-test (M = 43, SD = 29 nmol/L), *t*(53) = 11.36, *p* < 0.001, but did not differ significantly between groups. The increase in vitamin D knowledge from time 1–3 was significantly higher in the intervention than control group (*t*(88) = 2.26, *p* = 0.03). The intervention group (M = 3.52, SE = 0.13) had higher overall perceived importance of vitamin D supplementation than the control (M = 3.16, SE = 0.12), *F*(1, 88) = 4.38, *p* = 0.04, η_p_^2^ = 0.05.

**Conclusions:**

Although recommendations suggest blood 25(OH)D_3_ concentrations of ≥50-75 nmol/L, vitamin D status was below national recommendations. While participating in an intervention did not improve vitamin D status, it led to increased vitamin D intake, knowledge and perceived importance of supplementation.

**Trial registration:**

ClinicalTrails.gov registration #: NCT02118129.

**Electronic supplementary material:**

The online version of this article (doi:10.1186/s12966-016-0443-1) contains supplementary material, which is available to authorized users.

## Background

Vitamin D is crucial for bone health, including the prevention of rickets in children and osteomalacia in adults [1]. Sufficient serum vitamin D concentrations also may be protective against a range of disease states, including cancer, cardiovascular disease, diabetes and multiple sclerosis, and may enhance the immune system [2]. UVB is generally insufficient for subcutaneous production of vitamin D_3_ during the winter months in Canada, which is generally above 42° latitude [3, 4]. Further, individuals with darker skin pigmentations have a higher concentration of melanin in their skin, placing them at higher risk for vitamin D insufficiency [5]; this makes vitamin D particularly important for non-Caucasian individuals, including immigrants to Canada. Serum-hydroxy vitamin D (25(OH)D_3_) concentrations are regarded as the best measure of vitamin D status [6]; in 2010 the Institute of Medicine (IOM) published recommendations for vitamin D intake corresponding to a serum 25(OH)D_3_ concentration of 50 nmol/L [1]. More recent recommendations suggest blood concentrations of ≥75 nmol/L for the maintenance of optimal health [7, 8]. Poor vitamin D status is an important issue among young adults, since peak bone mass is reached before age 30 and cannot be significantly increased afterwards [9]. Unfortunately, many young adults are not meeting vitamin D recommendations. The 2012–2013 Canadian Health Measures Survey (CHMS) indicated that young adults aged 18–25 had a mean plasma vitamin D concentration of 60 nmol/L (95 % CI: 52.4-67.7) [10]. Given that young adulthood is a critical period for the development of long-term health behaviours [11], the formation of healthy habits, including adequate intake of vitamin D for the formation of peak bone mass, is crucial [12].

Few studies have examined what young adults know about vitamin D [13], or how to target this group with regards to increasing vitamin D status. In previous qualitative research, the authors found that young adults aged 18–25 were not generally worried about bone health, since potential consequences were perceived as being too distant to be a present concern [14]. Analysis of data from focus groups identified several themes related to engaging young adults about the importance of vitamin D, one of which was the importance of immediate, personally relevant information [14]. The concept of using a mobile app to track personal vitamin D intake emerged from these focus group discussions [14] and was used to inform the current study, which utilizes an online survey platform and the mobile Vitamin D Calculator app (VDC-app) [15].

The use of a web-based platform is not new to health research; several studies have used online surveys to examine health behaviours such as nutrition and smoking [16–19]. The growing popularity of smartphones [20] and health apps [21] have led to the increasing incorporation of mobile health apps in research [22, 23]. Mobile technology holds promise as a tool with which to engage young adults on health and nutrition issues [24], especially since 18-29-year olds use mobile apps to look up health information more frequently than adults of other ages [25]. In addition, although a few interventions have aimed to increase vitamin D and/or calcium intake in younger (i.e., non-elderly) populations [26–29], none have included young adults of both genders, or incorporated mobile apps. Thus, the current study used an online intervention involving a mobile app to target vitamin D intake, status, knowledge and perceptions among young men and women aged 18–25.

### Study objectives

#### Primary, impact objective

1. To determine whether an intervention involving the use of the mobile VDC-app produces changes in intake, knowledge, and/or perceptions of vitamin D among this sample of young adults.

#### Secondary, outcome objective

2. To determine blood concentrations of 25(OH)D_3_ at baseline and post-intervention.

## Methods

### Participants & recruitment

The sample consisted of 90 adult men and women aged 18–25 years. The study was advertised as a “Healthy Living Study” and did not specifically mention vitamin D. Participants were recruited during fall 2014 using poster and online advertisements in Guelph and throughout Ontario. In order to be eligible for the study, participants were required to own an iPhone/iPad/iPod Touch and to be: 18–25 years old, fluent in English, and currently living in Ontario. Participants were randomly assigned to either the intervention or control group using a single blind technique; the primary student investigator recruited participants and assigned them to groups based on a spreadsheet that was sequentially numbered with subject ID numbers and group allocations. Non-Caucasians tend to have a higher concentration of melanin and thus darker skin pigmentations, increasing their risk for vitamin D insufficiency [5]. Thus, quota sampling was conducted via email-screening to ensure that approximately half the sample identified as non-Caucasian, and that there were equal numbers of men and women. Finally, this study was conducted during the fall/winter months (September 2014 - March 2015) in order to focus the intervention on the importance of dietary vitamin D [3] intake during a period with insufficient UVB exposure for subcutaneous production of vitamin D [4]; the approximate latitude of Guelph, Ontario is 43.55° [30].

### Study design

This study was a randomized, controlled trial and followed a pre-post intervention and control group design. Vitamin D knowledge and perceptions were examined through online surveys administered to all participants at 3 time-points; vitamin D intake was measured using a food frequency questionnaire (FFQ) at pre- and post-test. The intervention group also received an online intervention which was offered to the waitlist-control group upon study conclusion (see Procedure). Finally, vitamin D status (i.e., blood vitamin D_3_ concentration; 25(OH)D_3_) was measured at pre- and post-test using blood spot tests. The blood spot test method has been highly correlated with serum 25(OH)D_3_ in previous research [31, 32] and was made optional to decrease self-selection bias (i.e., to avoid eliminating potential participants who were interested in study participation but who were not comfortable taking blood tests).

**Fig. 1:**
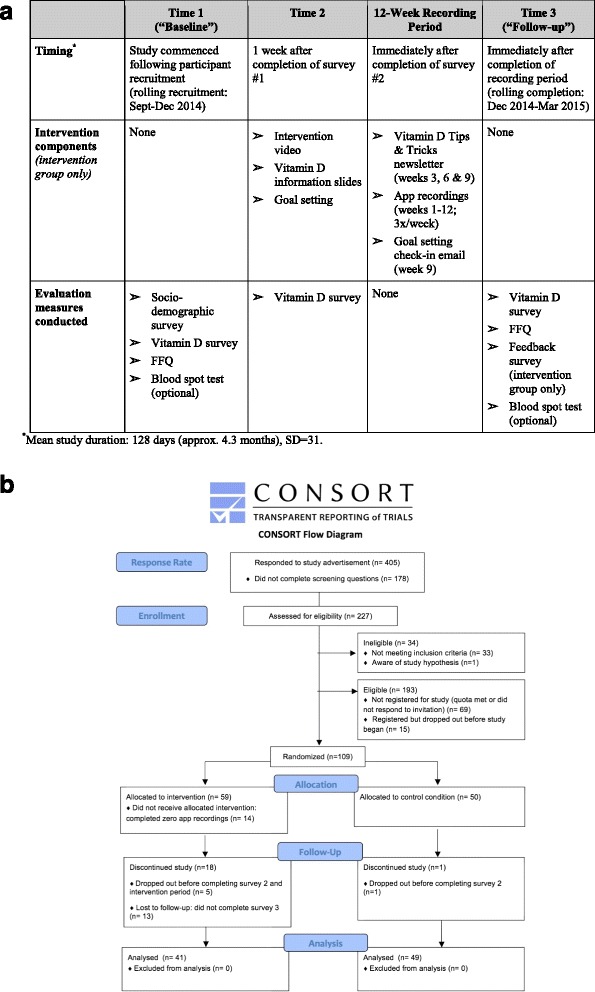
Intervention study design and flow. (**a**) Study design; (**b**) CONSORT flow diagram

The study was grounded in the Theory of Planned Behavior (TPB) [33] and Prototype Willingness Model (PWM) [34]; wherein factors including past behaviour, behavioural expectations, norms, perceived behavioural control, and intentions lead to changes in behaviour (i.e., vitamin D intake). A more detailed description and analysis of this theoretical framework is presented elsewhere (AUTHORS; under review; Health Education & Behavior) [35]. The study, including all online surveys, intervention materials and VDC-app recordings was pilot tested with 5 participants. The study received approval from the University of Guelph Research Ethics Board (#14MY027). Fig. 1 outlines the study design (1a) and CONSORT diagram (1b). The CONSORT (2010) checklist is available in Additional file 1 [36].

### Procedure

#### Time 1

After expressing interest in the study, participants were contacted and screened via email. Eligible participants received the link to the first online survey and were asked to complete it within one week. Participants completed an online consent form, followed by the measures included in the first online survey, which included a socio-demographic questionnaire, Vitamin D Survey (identical at all 3 time-points), and a FFQ measuring vitamin D intake. The online survey was administered via LimeSurvey version 1.91+ (LimeSurvey Project, Hamburg, Germany, Carsten Schmitz, 2012); data were stored on a secure SSL-enabled server at the University of Guelph. Participants were given the choice to participate in the blood test component of the study, and were told that the test measured blood levels of “specific nutrients” (i.e., no mention of vitamin D). Participants received a blood spot test kit by mail or pick-up. The kit included an addressed, postage-paid return envelope, and instructions to administer the blood test and to return it within one week. Baseline blood tests were completed between September-December 2014.

#### Time 2

One week later (or upon return of blood test), all participants were emailed a link to the second online Vitamin D Survey, which included the Fitzpatrick Skin Type questionnaire.

#### Intervention (12-week duration)

For intervention participants only, the survey link sent at time 2 initialized a video and educational slides about vitamin D. In the intervention video (2:08 min), a Registered Dietitian from a Canadian news agency explained key facts about vitamin D [37]. Immediately after the video, additional facts about vitamin D were displayed on information slides. The video and information slides served to educate participants on eight key points (i.e., learning goals) related to vitamin D; see list of points in Table 1. The information slides were followed by a screen instructing participants to set a ‘SMART’ goal (SMART *= S*pecific, *M*easurable, *A*chievable, *R*ealistic, and *T*ime-related) related to vitamin D; see wording in Additional file 2. Upon completion of the second online Vitamin D Survey, intervention participants received email instructions outlining how to download and use the free VDC-app on their device. Participants were given a choice of two weekdays and one weekend day and instructed to record their dietary intake and time spent in sunlight 3 days/week over the next 12 weeks (36 recording days). They were provided with a unique subject ID and their Fitzpatrick Skin Type to enter into the app. The VDC-app was previously validated as a measure of dietary vitamin D intake and classification [38]. It allows users to enter intake of vitamin D and calcium-containing foods, beverages, and supplements, as well as time spent in sunlight. The user’s postal code is entered and automatically links to the daily UV forecast by Environment Canada. Users receive immediate feedback regarding their estimated vitamin D and calcium intake in relation to current recommendations, and a pie chart illustrates the user’s consumption of vitamin D or calcium from various dietary sources. A more detailed description of the app is presented elsewhere [38]. App data were stored and downloaded from a secure online database at the University of Guelph.

**Table 1:** Intervention information points (i.e., learning goals)*

Information Point	Description of information presented
1.	▪ The key dietary sources of vitamin D (i.e., fish, cow’s milk/fortified milk alternatives)
2.	▪ Vitamin D is synthesized in the skin from solar UV exposure after approximately 15–30 min of exposure to bare skin
3.	▪ Our bodies cannot make vitamin D from the sun in the fall/winter months in Canada ▪ The importance of taking vitamin D supplements and/or consuming adequate amounts of vitamin D from foods and beverages in the fall/winter months in Canada
4.	▪ The recommended intakes for adults (IOM: RDA = 600 IU; UL = 4,000 IU) and the fact that higher intakes are suggested by some vitamin D researchers (≥1000 IU/day) ▪ Many Canadians fall short of these recommendations
5.	▪ Why we need vitamin D (i.e., to absorb calcium, effects on bone health) ▪ Brief summary of other potential health outcomes associated with vitamin D insufficiency
6.	▪ Vitamin D_3_ is the form we receive from the sun and most supplements
7.	▪ Factors that affect vitamin D status, including: age, sex, weight, cloud cover, clothing, sunscreen, season/UV index, location and skin pigmentation
8.	▪ Skin pigmentation/ethnicity affects vitamin D status ▪ Those with darker skin pigmentations may be at risk of vitamin D insufficiency

^*^Intervention video available at: http://www.theglobeandmail.com/life/life-video/video-tips-for-getting-enough-vitamin-d-in-your-diet-this-winter/article17385017/

On weeks 3, 6 and 9, intervention participants received an email that reminded them to complete their recordings, as well as a vitamin D newsletter in PDF format. The 3 newsletters were developed by the researchers and included key facts, recipes and tips about vitamin D (see Additional file 2). On week 9, intervention participants were asked to respond to two goal-setting questions by email. The results of a previous qualitative study examining strategies to communicate online nutrition information to Canadians aged 18–25 years [14] was used to inform the current intervention.

#### Time 3

At the end of week 12, all participants received a link to the third online survey, which included the Vitamin D Survey and FFQ. A short intervention feedback survey was included for intervention participants only. After completing the online survey, participants completed the second optional blood test. With one exception, post-intervention blood tests were completed between December 2014-March 2015. Throughout the study, participants received email reminders if they had not completed a study component within the requested timeframe. After completion of the third online survey (or upon return of blood test), participants were mailed compensation and a debriefing form that explained the study purpose. Compensation to all participants was a $50 grocery gift card; those who participated in the blood tests received a $70 gift card.

#### Control participants

Control participants did not participate in the intervention nor were they informed of the app during the study period. The following data were collected from control participants: socio-demographic survey and Fitzpatrick Skin Type Questionnaire (time 1), vitamin D survey (times 1, 2, 3), FFQ (times 1 and 3), and optional blood spot test (times 1 and 3); see Fig. 1a. The debriefing form instructed waitlist-control participants to email the researchers if they wished to view the intervention video and/or download the VDC-app.

### Measures

The following measures were completed by all participants; please see Additional file 2 for item wording and scales for all measures. *Socio-demographic Survey.* This survey was developed by the researchers and collected demographic information, including age, gender, ethnicity, height, weight, education level, and student status. *Vitamin D Survey.* This survey was developed by the researchers and consisted of 41 questions that examined vitamin D intake habits, knowledge and perceptions, and items adapted for vitamin D based on the TPB [33] and PWM [34]. As mentioned above, analysis of these theoretical items is presented elsewhere (AUTHORS; under review; Health Education & Behavior). The Vitamin D Survey was administered at all three time-points in order to test influence of the intervention on vitamin D knowledge and perceptions after two distinct phases of the intervention: (1) vitamin D video and information slides; (2) newsletters and use of VDC-app. A 3-item subscale was used to measure the perceived importance of taking vitamin D supplements: *“From a health perspective, it is important for me to…”* (1)*“take vitamin D supplements in the spring/summer months in Canada”,* (2)*“take vitamin D supplements in the fall/winter months in Canada”,* and (3) *“regularly take vitamin D supplements or multivitamins containing vitamin D”* (*1 = strongly disagree; 5 = strongly agree*)*. Fitzpatrick Skin Type Questionnaire.* This survey was developed and validated by Fitzpatrick [39] to classify six skin types according to reactivity to the sun. *Blood spot test kit.* Kits consisted of a lancet, blood spot test card, alcohol swab, bandage and instructions. Test kits were provided by GrassrootsHealth [40] and were analyzed using liquid chromatography–tandem mass spectrometry by ZRT Laboratory to determine blood 25(OH)D_3_ concentrations [41]. *FFQ.* The FFQ has been previously validated to assess self-reported vitamin D and calcium intake [42]. It includes one open-ended question on nutritional supplement use and 37 items relating to specific foods containing vitamin D and/or calcium. For each item, respondents select a serving size and frequency of consumption. Serving sizes are described within, and correspond to typical household measures for each item [42]. *Goal-Setting and Feedback Survey (intervention participants only*). Intervention participants recalled and assessed achievement of their SMART goal, indicated how many newsletters they read and rated how much the various intervention components influenced their vitamin D-related behaviours. Participants rated their liking and usability of the app, and answered three open-ended feedback questions.

### Data analysis

#### Variable coding

Body mass index (BMI) was calculated using reported height and weight, and the World Health Organization international classification of BMI cut-offs [43] determined weight classification. Ethnicity was recoded into a binary variable (0 = Caucasian, 1 = non-Caucasian) where participants who identified as Caucasian/White or European were considered Caucasian, and all other participants were considered non-Caucasian. Fitzpatrick Skin Types were determined according to the Fitzpatrick Skin Type Questionnaire [39]. The number of recordings participants submitted using the VDC-app were summed to form an “app use” score (control participants received a score of zero). A “vitamin D knowledge” score (max. score = 9) was computed by summing the score of nine items from the Vitamin D Survey (see Additional file 2). The 3 items measuring perceived importance of vitamin D supplements were significantly correlated (*p* < 0.001) and showed good internal reliability using Cronbach’s alpha (α = 0.81)*. Statistical analyses.* Statistical analyses were performed using SPSS version 22.0 (SPSS Statistics, Armonk, NY, IBM Corp., 2013). Differences between the control and intervention group on socio-demographic factors and key outcomes at baseline were tested using student’s *t*-tests, chi-squared tests (*χ* ^2^) and univariate analyses of variance (ANOVA) for binary, categorical and continuous outcomes, respectively. Bivariate Pearson’s correlations were used to test for significant associations between variables. Univariate ANOVAs were used to test for differences between the control and intervention groups in the change in vitamin D intake and status. A power calculation conducted a priori indicated that a sample size of 84 participants (*n* = 42 per group) would provide 80 % power to detect a 5 % difference in intake (approximately 8 IU) between groups. Repeated-measures ANOVAs were used to test for differences between the control and intervention group (between-subjects factor) in vitamin D knowledge and perceptions at the three time-points (within-subjects factor). Paired samples *t*-tests confirmed significant differences in outcomes from pre- to post-intervention. FFQ data were analyzed using a Microsoft Excel template provided by the developer (S. Whiting, personal communication, May 16, 2014).

## Results

The final sample consisted of 90 adults aged 18–25 (M = 22, SD = 2.0) years; 42 % men (*n* = 38) and 58 % women (*n* = 52). Forty-one percent of the sample identified as non-Caucasian. Detailed sample characteristics are listed in Table 2. As shown in Fig. 1b, the original sample was 109 men and women; 17 % (*n* = 19) were lost to follow-up. Attrition rates did not differ significantly between Caucasian and non-Caucasian participants. Men were significantly more likely to leave the study compared to women (27 % vs. 9 %), *F* = 30.03, *t*(107) *=* 2.55, *p* = 0.01. Individuals in the intervention group were significantly more likely to drop out compared to those in the control group (31 % vs. 2 %), *F* = 152.93, *t*(107) *=* 4.18, *p* < 0.001. Of those who dropped out, two participants had technical difficulties with their Apple device and the remainder stopped responding to survey reminders or emails. Of those who remained in the study at time 3 (*n* = 90), the mean length to follow-up was 128 days (SD = 31). Student’s *t-*tests, *χ* ^2^ and ANOVAs indicated that the intervention and control group did not differ significantly on any of the following variables: gender, age, ethnicity, BMI, education level, employment, student status, supplement use, being employed in/studying health or nutrition, mean daily vitamin D intake or vitamin D_3_ concentrations (*p* > 0.05). Ethnicity (Caucasian vs. non-Caucasian) and Fitzpatrick Skin Type were significantly correlated, *r*(88) = 0.47, *p* < 0.001.

**Table 2:** Sample characteristics of participants participating in vitamin D intervention study (*n* = 90)

Variable	Intervention (*n* = 41) % (n)	Control (*n* = 49) % (n)	Total (*n* = 90) % (n)
Sex			
Male	34 % (14)	49 % (24)	42 % (38)
Female	66 % (27)	51 % (25)	58 % (52)
Age			
18-19	24 % (10)	8.1 % (4)	16 % (14)
20-21	20 % (8)	39 % (19)	30 % (27)
22-23	32 % (13)	22 % (11)	27 % (24)
24-25	24 % (10)	31 % (15)	28 % (25)
Ethnicity			
White/Caucasian	46 % (19)	55 % (27)	51 % (46)
Asian, South Asian, Southeast Asian	17 % (7)	12 % (6)	14.5 % (13)
European	7 % (3)	8 % (4)	8 % (7)
Middle Eastern/Arab	7 % (3)	2 % (1)	4 % (4)
African/Caribbean	7 % (3)	2 % (1)	4 % (4)
Mixed ancestry	5 % (2)	6 % (3)	6 % (5)
Other ethnicity (Aboriginal, Latin/Central American, Filipino, other)	10 % (4)	14 % (7)	12 % (11)
Highest level of education			
Some high school, or high school diploma	24 % (10)	10 % (5)	17 % (15)
Some college, college diploma or professional certificate	12 % (5)	14 % (7)	13 % (12)
Some university, or undergraduate degree	56 % (23)	63 % (31)	60 % (54)
Some graduate school, or graduate degree	7 % (3)	12 % (6)	10 % (9)
Student status			
Currently a student	76 % (31)	61 % (30)	68 % (61)
BMI Classification			
Underweight (<18.5)	5 % (2)	6 % (3)	6 % (5)
Normal weight (18.5-24.9)	70 %(28)	55 % (27)	62 % (55)
Overweight (25.0-29.9)	20 % (8)	18 % (9)	19 % (17)
Obese (≥30)	5 % (2)	20 % (10)	13 % (12)

Note: Student’s *t-*tests, *χ* ^2^ and ANOVAs indicated that the intervention and control group did not differ significantly on any of the following variables: gender, age, ethnicity, BMI, education level, employment, student status, supplement use, being employed in/studying health or nutrition, mean daily vitamin D intake or vitamin D_3_ concentrations (*p* > 0.05)

### Vitamin D intake

Total vitamin D intake from the FFQ was positively skewed; the natural logarithm transformation [ln(x)] was used to correct for non-normality. Results from transformed data are reported for subsequent analyses; for ease of interpretation, means and standard deviations (SD) are reported for untransformed data in Table 3. Mean vitamin D intake of the full sample at baseline did not differ significantly by study group, age, gender, ethnicity, education, BMI or supplement use (*p* > 0.05 for all). Vitamin D intake was significantly higher among men than women at post-test (*t*(42.74) = 2.14, *p* = 0.04; Table 3). App use was significantly correlated with education level (*r*(39) = 0.31, *p* = 0.048); thus, these two variables were entered as covariates in the ANOVA. Results indicated that after adjusting for gender and education, there was a significant effect of study group on the change in total mean vitamin D intake from pre- to post-test, *F*(1, 85) = 4.09, *p* = 0.046, η_p_^2^ = 0.05; whereby the mean vitamin D intake of intervention participants increased more than that of control participants (+308 IU vs. 131 IU, respectively).

**Table 3:** Mean (SD) vitamin D intake (IU/day) among participants at pre- and post-test (M = 128 days, SD = 31)^*^

Pre-test	All participants	Study Group	
	(*n* = 90)	Intervention (*n* = 41)	Control (*n* = 49)
Food & beverages	229 (245)	203 (145)	250 (304)
Supplements	178 (396)	**191 (473)** ^**b**^	168 (322)
Total (all sources)	**407 (460)** ^**a**^	**394 (494)** ^**c**^	418 (434)
Post-test	All participants	Study Group	
	(*n* = 89)^**+**^	**Intervention** (*n* = 41)	**Control** (*n* = 49)^**+**^
Food & beverages	247 (280)	244 (342)	249 (240)
Supplements	369 (619)	**458 (657)** ^**b**^	294 (582)
Total (all sources)	**619 (655)** ^**a**^	**702 (714)** ^**c**^	549 (598)

^**+**^Note: At post-test, *n* = 48 for control group and *n* = 37 for males in totals for foods/beverages and total vitamin D, due to a missing data point

*Significant differences are indicated in bold with subscript lettering. No significant difference found in mean vitamin D intake between study groups at pre- or post-test, *p* > 0.05

^a^Total mean daily vitamin D intake increased significantly among the full sample from pre- to post-test, *t*(88) = 5.37, *p* < 0.001

^b^Supplemental vitamin D intake increased significantly from pre- to post-test in intervention group, *t*(40) = 3.37, *p* < 0.01 but not control group, *p* > 0.05

^c^Total mean vitamin D intake increased significantly from pre- to post-test in intervention group, *t*(40) = 2.78, *p* < 0.01 but not control group, *p* > 0.05

### Vitamin D status

Fifty-eight participants completed the optional blood spot test at baseline. Baseline 25(OH)D_3_ concentrations did not vary significantly by age, study group, gender, education, BMI or Fitzpatrick Skin Type (*p* > 0.05). Baseline 25(OH)D_3_ concentration (nmol/L) was significantly higher among Caucasians (M = 31.8, SD = 2.73) than non-Caucasians (M = 19.3, SD = 2.32), *t*(56.7) = 3.49, *p* = 0.001. The baseline vitamin D status of this sample was classified in relation to the IOM’s thresholds for deficiency (25–30 nmol/L) and sufficiency for bone health (50 nmol/L) [1], and the recommendation of 75 nmol/L for optimal health [4]. More than half (56.9 %; *n* = 33) of participants were vitamin D deficient (<25 nmol/L) and 70.7 % (*n* = 41) were below 30 nmol/L. The vast majority (91.4 %; *n* = 53) had concentrations below 50 nmol/L, and all participants (100 %; *n* = 59) failed to meet the 75 nmol/L cut-off. Participants who completed versus those who declined to complete blood tests did not significantly differ in age, study group, gender, ethnicity, education, or BMI (*p* > 0.05). Blood 25(OH)D_3_ data were positively skewed at post-test. The natural logarithm transformation [ln(x)] corrected normality of this variable; log-transformed data are reported for subsequent analyses relating to post-test data. Means and standard deviations are reported for untransformed data in Table 4. Blood 25(OH)D_3_ concentrations were significantly positively correlated with total mean vitamin D intake from the FFQ at post-test (*r*(53) = 0.46, *p* < 0.001), but not at pre-test (*p* > 0.05). A univariate ANOVA indicated no significant effect of study group on the change in 25(OH)D_3_ concentrations from pre- to post-test (*p* > 0.05). Subsequent adjusted models indicated that study group remained non-significant after adjusting for gender, age, education, BMI, Fitzpatrick Skin Type, physical activity, baseline 25(OH)D_3_, supplement use, and seasonality (i.e., month of blood pre- and post-test), *p* > 0.05 for all. Study group also remained non-significant (*p* > 0.05) after adjusting for ethnicity, which had a significant main effect (*F*(1,51) = 7.07, *p* = 0.01, η_p_^2^ = 0.12).

**Table 4:** Mean (SD) blood 25(OH)D_3_ concentrations (nmol/L) among participants at pre-test (Sept-Dec) and post-test (Dec-Mar)^*^

	All participants	Study group	
		Intervention	Control
PRE-TEST	(*n* = 59)	(*n* = 25)	(*n* = 34)
	**27 (16)** ^**a**^	28 (16)	26 (15)
POST-TEST	(*n* = 56)	(*n* = 23)	(*n* = 33)
	**43 (28)** ^**a**^	46 (31)	42 (27)

*Significant differences are indicated in bold with subscript lettering. No significant differences found between study groups at pre-test or post-test, *p* > 0.05

^b^Mean 25(OH)D_3_ of the full sample increased significantly from pre- to post-test, *t*(53) = 11.36, *p* < 0.001

### Perceived importance of vitamin D supplementation

Six items relating to the perceived importance of vitamin D-related behaviours were measured; mean agreement over time is listed in Table A.1 (see Additional file 3). As described in Data Analysis, a 3-item measure was used to assess perceived importance of vitamin D supplementation. The intervention and control groups did not differ on responses to this item at baseline (*p* > 0.05). A repeated-measures ANOVA indicated significant main effects of time [*F*(1.83, 161.23) = 3.34, *p* = 0.04, η_p_^2^ = 0.04] and study group, *F*(1, 88) = 4.38, *p* = 0.04, η_p_^2^ = 0.05, whereby agreement increased in both groups over time, and the intervention group had higher agreement overall (M = 3.52, SE = 0.13) compared to the control group (M = 3.16, SE = 0.12).

### Vitamin D knowledge

Baseline vitamin D knowledge did not differ significantly by study group and app use was not significantly correlated with baseline vitamin D knowledge (*p* > 0.05 for all). Repeated-measures ANOVAs examined the change in vitamin D knowledge (max. score = 9) across time. As shown in Table 5, the effects of time and study group were significant in 2-way and 3-way models. Vitamin D knowledge was higher overall in the intervention (M = 5.01, SE = 0.22) than the control group (M = 4.26, SE = 0.20) across time. Knowledge among intervention participants increased more from time 1 to 2 (+1.88) than among control participants (+0.19), and the net increase in mean vitamin D knowledge from time 1 to 3 was significantly higher in the intervention (+0.91) than control group (+0.25), *t*(88) = 2.26, *p* = 0.03. After adjusting the model for app use (*F*(1,87) = 4.03, *p* = 0.048, η_p_^2^ = 0.04), the effect of study group became non-significant (*p* > 0.05). In order to explore this relationship, an additional analysis was conducted among intervention participants only (*n* = 41). Intervention participants were classified into two groups: no/low app use (<20 recordings; *n* = 24) or frequent app use (≥20 recordings; *n* = 17). Vitamin D knowledge of the two groups did not differ at baseline (*p* > 0.05), but was significantly different at time 2 (*t*(39) = −2.15, *p* = 0.04) and time 3 (*t*(39) = −3.39, *p* < 0.01). A 2-way ANOVA was conducted to examine the change in vitamin D knowledge during the intervention (time 2–3), while adjusting for time 2 knowledge. Significant main effects of time 2 knowledge (*F*(1,38) = 16.35, *p* < 0.001, η_p_^2^ = 0.30) and frequency of app use (*F*(1,38) = 6.01 *p* < 0.02, η_p_^2^ = 0.14) were found. The vitamin D knowledge of both groups decreased from time 2–3, but frequent app users had higher scores at both time-points and a smaller decrease in vitamin D knowledge (−0.69) than those who used the app infrequently/not at all (−1.17). In sum, more frequent app use counteracted the decrease in vitamin D knowledge observed from time 2–3.

**Table 5:** Results of repeated-measures ANOVAs measuring change in vitamin D knowledge across intervention study time-points^a^

	Study group			Time			Study group x time		
ANOVA	***F*** **(** ***df)***	***P***	**η**_p_^**2**^	***F*** **(** ***df*** **)**	***P***	**η**_p_^**2**^	***F*** **(** ***df)***	***P***	**η**_p_^**2**^
**2-way**	7.14 (1, 88)	**<0.01**	0.08	47.29 (1, 88)	**<0.001**	0.36	32.53 (1, 88)	***p*** **< 0.001**	0.27
**3-way**	6.13 (1, 88)	**0.02**	0.07	25.52 (2, 176)	**<0.001**	0.23	17.03 (2,176)	***p*** **< 0.001**	0.16

^a^Significant differences (*p* < 0.05) are indicated in bold. Two-way ANOVA examined changes from time 1 to 2; three-way ANOVA examined changes across times 1, 2 & 3

### Adherence to intervention components

The following section outlines adherence to individual components of the intervention, as part of a process evaluation.

#### Use of Vitamin D calculator

About 66 % (*n* = 27) of intervention participants submitted at least one app recording to the online database. Including the 34 % (*n* = 14) of intervention participants who did not submit app recordings, a mean of 14 (SD = 16) recordings were submitted. Among those who submitted recordings, the average number of submissions was 21 (SD = 15; range = 1–63). Number of app submissions (‘app use') did not differ significantly between men and women, *p* > 0.05. App use was not correlated with change in mean vitamin D intake or status (*p* > 0.05), but was significantly positively correlated with education level, *r*(39) = 0.31, *p* = 0.048.

#### Newsletters

Over half (51 %, *n* = 21) of intervention participants reported reading one or two vitamin D newsletters, 29 % (*n* = 12) reported reading all three, and 20 % (*n* = 8) read none.

#### Goal Setting

All intervention participants (*n* = 41) set a SMART goal during survey 2. Over half (54 %, *n* = 22) responded to the goal-setting check-in email sent during the recording period. Of those who responded, 68 % (*n* = 15) indicated that seeing their vitamin D results in the VDC-app led to increased goal commitment, and 59 % (*n* = 13) indicated that it led them to modify or change their goal. The follow-up survey indicated that 39 % (*n* = 16) of intervention participants remembered their SMART goal at time 3, and 34 % (*n* = 14) indicated that they altered their goal after receiving personal vitamin D feedback from the app.

### Intervention feedback

Detailed results of the feedback survey are shown in Tables A.2 and A.3 (see Additional file 3). About half of intervention participants reported that they liked using the VDC-app “somewhat” or “very much” (46 %), and that it was “somewhat” or “very” easy to use (54 %). App use was significantly positively correlated with liking the app, *r*(39) = 0.57, *p* < 0.001 and reported ease of use, *r*(39) = 0.43, *p* < 0.01. Qualitative data were provided to the app developer.

## Discussion

### Baseline findings

This study had many important findings, the first being that vitamin D status was low at baseline (M = 27 nmol/L), falling well below the recommended concentrations of 50 nmol/L for bone health [1] and 75 nmol/L for optimal health and disease prevention [7, 8]. The mean 25(OH)D_3_ concentration in our sample was somewhat comparable to that of a previous study examining adults aged 18–30 living in Toronto, Ontario (M = 39.4 nmol/L) [44]. However, it was much lower than the national average of approximately 60 nmol/L reported among 18–25 year olds in the CHMS. Given that the CHMS participants were sampled from August 2009-November 2011 [45], the discrepancy can likely be attributed to blood sampling year-round rather than during fall/winter, as in the current study. Further, we conducted quota sampling to ensure that roughly half the sample was non-Caucasian. The relatively large prevalence of non-Caucasians in our final sample (41 %) may thus have contributed to our lower 25(OH)D_3_ concentration at baseline, since individuals with darker skin pigmentations tend to have lower blood vitamin D concentrations [46]. Interestingly, vitamin D status did not differ significantly by BMI or Fitzpatrick Skin Type, despite previous findings suggesting that vitamin D status tends to be lower among individuals who are obese and/or have darker skin pigmentation [46]. However, given that ethnicity and Fitzpatrick Skin Type were significantly correlated, ethnicity (Caucasian vs. non-Caucasian) may have acted as a proxy for skin pigmentation.

#### Intervention efficacy

Firstly, the intervention administered herein led to a modest increase in vitamin D intake. Results indicated that after adjusting for gender and education, study group had a significant effect on the change in vitamin D intake from pre- to post-intervention. Specifically, the mean vitamin D intake of intervention participants increased more than that of control participants. Mean vitamin D intake from supplements increased significantly by 267 IU/day among intervention participants, while a non-significant increase was observed in the control group. The increase in total daily vitamin D intake (food + supplements) was thus approximately 43 % greater in the intervention (+308 IU) than the control group (+131 IU). The additional 177 IU/day vitamin D consumed by intervention participants is roughly equivalent to an extra 1¾ cups of milk or ½ to 1 serving of oily fish per day [47], an increase we feel is clinically relevant. These results suggest that the intervention model administered herein led to improvements in total vitamin D intake, largely due to increased supplemental vitamin D. These findings contrast with those of a previous study by Bohaty et al. who did not find significant increases in vitamin D intake after an educational intervention involving a slideshow, group discussion, information packet, and follow-up call [26]. The additional components of our intervention design (i.e., goal setting, self-monitoring and personal feedback via mobile app) may have contributed to the observed differences in vitamin D intake. Further, as the Bohaty study consisted of 80 females, our slightly larger, mixed-gender sample may have also contributed to differing results.

Secondly, blood vitamin D concentrations in our sample improved significantly from pre-test (27 nmol/L) to post-test (43 nmol/), but did not differ significantly between groups. Two assumptions regarding this finding are worth noting: (1) blood vitamin D concentrations at pre-test did not appear to be inflated by carryover from summer UVB (i.e., no seasonality effect) and (2) concentrations in the winter are expected to be equal to, or even lower than those in the fall, which was the opposite of what was observed. Given that the change in status was not significantly different between the two groups, we can conclude that the intervention alone did not appear to significantly influence 25(OH)D_3_ concentrations. The lack of difference between groups may be explained by inadequate statistical power, and the fact that a multitude of environmental factors affect circulating vitamin D concentrations [4]. It is worth noting that the amount of dietary vitamin D required to raise blood vitamin D concentrations (i.e., dose–response curve) differs widely across individuals [48], thus even a consistent increase in intake would not uniformly raise serum levels.

Thirdly, an analysis of survey measures indicates that participation in the intervention led to improved perceptions and knowledge of vitamin D. Overall, the intervention group agreed more strongly with the importance of taking vitamin D supplements than the control group, suggesting that the intervention had the intended effect. Vitamin D knowledge increased significantly only in the intervention group. Given that this increase was largest at time 2 (i.e., after the educational slides), the intervention video and information slides seem to have had the greatest effect on vitamin D knowledge. These findings align with those of a previous study that found increased knowledge of vitamin D after an educational intervention [26]. Further, the observed decline in vitamin D knowledge from time 2 to time 3 was more drastic for intervention participants who used the app infrequently or not at all, suggesting that more frequent use served to temper the decrease in vitamin D knowledge that occurred over time. Finally, higher education was associated with more frequent app use, similar to previous research indicating that individuals with higher education levels were more likely to adhere to a dietary intervention [49].

In assessing efficacy of the intervention, it is important to note that adherence was less than ideal: while 36 recordings indicated perfect adherence to the app, over a third of participants submitted none. Among those who participated, the average rate of app submissions was 58 % (M = 21 recordings). Although no gold standard exists for measuring adherence to different health behaviour interventions [50], our adherence rates were comparable to those of other online dietary intervention programs. An online intervention aiming to prevent weight gain in undergraduate students reported an adherence rate of about 69 % in their internet intervention group [51], while an online intervention assessing vitamin D intake reported an online tutorial completion rate of 59 % [52]. Similar to our study, these authors reported lower retention rates in the intervention than control group [51, 52], suggesting that our findings are not atypical. This greater attrition and imperfect adherence to the intervention indicate that in real-world settings, effectiveness of the program may be limited to more motivated individuals.

Regarding intervention design, a review of the components included in e-Health interventions indicated that contact delivering behavioural change techniques was associated with greater success than simple email reminders [53]. Although our email reminders included behavioural techniques in the attached newsletters, the fact that participants had to open an attachment may have diminished their impact. On the other hand, a review of strategies to increase exposure to online behaviour change interventions targeting young adults [54] found that interventions that combined personalized feedback, reminders, and incentives had higher exposure. All three strategies were elements of the current intervention; our modest “exposure” rates (e.g., use of app, reach of newsletters) may have been lower had these elements not been included.

Finally, we observed “small” to “medium” effects of study group using partial eta squared [55] for two outcomes: change in vitamin D intake (η_p_^2^ = 0.05) and vitamin D knowledge (η_p_^2^ *=* 0.07). Previous meta-analyses have found small effect sizes overall (using Cohen’s *d* interpretations of effect) among online dietary behaviour change [56] and healthy eating interventions [57]. Although partial eta squared and Cohen’s *d* cannot be compared directly [55], it is notable that effect sizes for dietary interventions tend to be small but significant [57]. A systematic review [54] found that online health behaviour change interventions employing a greater number of behaviour change techniques tended to have greater exposure. Similarly, a meta-regression found that use of more self-regulation strategies was associated with larger intervention effects, and that interventions using self-monitoring plus at least one other technique were significantly more effective [57]. The current intervention utilized the following self-regulation strategies: goal setting, feedback, self-monitoring, and goal review/assessment; further, although intention formation was not required of participants, behavioural intentions were assessed at all three survey waves. In sum, our intervention model included many of the strategies cited above, which may have contributed to our small but significant effect sizes.

### Limitations

To begin, adherence to our intervention was moderate. Results indicated that lower adherence was associated with dislike of and difficulty using the app; these factors may have dissuaded some participants from regularly submitting recordings. Given that attrition was also higher among intervention participants, it is possible that some found three weekly app recordings to be too arduous. Secondly, the Vitamin D Survey used herein was developed for the current study. Given that vitamin D knowledge and perceptions were secondary outcomes (i.e., to vitamin D intake), externally validating this survey was beyond the scope of the study. To our knowledge, no validated vitamin D surveys exist; the vitamin D knowledge scale by Boland et al. [13] also was not externally validated. However, given that we observed between-group differences in vitamin D knowledge and perceptions, we feel that our survey was justified as a measure of intervention efficacy. Thirdly, we acknowledge the modest increases in vitamin D knowledge and intake observed in the control group. Although study materials and advertisements did not mention vitamin D, all participants completed online surveys containing vitamin D-related questions, which may have led to a “study effect” in which participants sought out vitamin D information. External factors may also have attributed to the increased intake in both groups, since vitamin D has recently been featured in the media [58–61]. Nevertheless, while both groups had equal access to the internet and other external factors, significant differences were observed between groups. This suggests that a genuine increase in vitamin D knowledge and intake occurred in the intervention group. Fourthly, we received additional funding later into the study design phase, which allowed us to add an optional blood testing component. Unfortunately, only 62 % of participants completed both optional blood tests, leading to decreased power to detect small between-group differences in 25(OH)D_3_ concentrations. Similarly, although our sample size calculation for vitamin D intake was based on the ability to detect a difference of 8 IU, this difference may not be considered clinically significant. Lastly, this study was conducted with a sample of 18–25 year olds in Ontario, Canada, 68 % of whom were students. Results cannot necessarily be generalized to other populations.

## Conclusions

An intervention that consisted of information conveyed through an online video, slides, and electronic newsletters, as well as dietary tracking through the mobile VDC-app was modestly successful at improving intake, knowledge and perceived importance of vitamin D, but not blood concentrations in a young adult population. Future researchers could adapt this intervention model to promote knowledge and intake of other nutrients without immediately apparent health consequences (e.g., calcium, omega-3) or health-protective behaviours. Vitamin D intake also increased somewhat among control participants after simply participating in surveys related to vitamin D. This finding suggests that a targeted public health campaign aiming to increase awareness of the importance of vitamin D might be sufficient to improve intake among young adults (i.e., without investing significant resources into individualized health interventions).

## Additional files

Additional file 1: CONSORT 2010 Checklist. (PDF 144 kb)
Additional file 2: Survey Materials. Intervention study survey materials and scoring. (PDF 991 kb)
Additional file 3: Additional Tables. Intervention study supplementary tables A.1, A.2 and A.3. (PDF 138 kb)

## Abbreviations

25(OH)D_3_: 1-25-dihydroxycholecalciferol, or serum vitamin D_3_ concentration
ANOVA: Analysis of variance
BMI: Body mass index
CHMS: Canadian health measures survey
FFQ: Food frequency questionnaire
IOM: Institute of Medicine
PWM: Prototype willingness model
SD: Standard deviation
SE: Standard error
TPB: Theory of planned behavior
