# Supplementary material for: A randomized, controlled trial evaluating the efficacy of an online intervention targeting vitamin D intake, knowledge and status among young adults
Source: Int J Behav Nutr Phys Act. 2016 Nov 11;13:116. doi: 10.1186/s12966-016-0443-1 (PMC5106840; doi:10.1186/s12966-016-0443-1)
Supplement: Additional file 2: — Survey Materials. Intervention study survey materials and scoring. (PDF 991 kb) [file 12966_2016_443_MOESM2_ESM.pdf]

Additional File 1: Survey Materials

Intervention Study Demographic Questionnaire

UNIVERSITY of GUELPH

1) What is your gender?
   1. Male
   2. Female
   3. Prefer not to say

2) What is your current age? ________ years

**If participant is above or below 18-25 years, online survey should not allow them to continue.*

3) Which of these best describes your racial or ethnic background?
   1. White/Caucasian
   2. Japanese, Chinese, or Korean
   3. South East Asian
   4. South Asian
   5. African or Caribbean
   6. European
   7. Middle-Eastern/Arab
   8. Latin American (including Central America)
   9. Filipino
   10. Aboriginal
   11. Mixed ancestry
   12. Other: ______________________
   13. Prefer not to say

4) Are you currently a student?
   1. No
   2. Yes
   3. Prefer not to say

5) What is the highest level of formal education you have achieved?
   1. Some high school
   2. High school diploma
   3. Apprenticeship/professional certificate

4. Some college
5. College diploma
6. Some university
7. Undergraduate university degree
8. Some graduate school
9. Graduate degree
10. Other (please specify): ______________________________________
11. Prefer not to say

6) What is your employment status?

1. Not employed
2. Employed part-time
3. Employed full-time
4. Prefer not to say

7) Have you ever taken a nutrition course?

1. Yes
2. No
3. Don't know/prefer not to say

8) Which of the following best describes who makes decisions regarding food purchases in your household?

1. I have complete responsibility
2. I have primary responsibility, but someone else (or several others) also contribute
3. I share responsibility equally with another person
4. Someone else has primary responsibility, but I also contribute
5. Someone else has complete responsibility
6. Other (please specify) ___________________

9) Is your current or past profession or program of study in a field related to ***health or nutrition?*** *(e.g. Nutrition, Health, Wellness, Human Biology, Medicine)*

1. Yes
2. No
3. Don't know/prefer not to say

10) Have you ever been diagnosed with osteoporosis, osteopenia, or low bone density?

1. Yes
2. No
3. Don't know/prefer not to say

11) Do you regularly wear a hijab or other type of veil that partially covers your face/head for cultural or religious reasons?

1. Yes
2. No
3. Don't know/prefer not to say

12) How many times a week, do you usually do 20 minutes of vigorous physical activity that makes you sweat or puff and pant? (For example, jogging, heavy lifting, digging, aerobics, or fast bicycling)

1. >3 times/week

2. 1-2 times/week

3. None

13) How many times a week, do you usually do 30 minutes of moderate physical activity or walking that increases your heart rate or makes you breathe harder than normal? (For example, mowing the lawn, carrying light loads, bicycling at a regular pace, or playing doubles tennis)

1. >5 times/week
2. 3-4 times/week
3. 1-2 times/week
4. None

14) How often do you engage in weight-bearing physical activity (such as lifting free-weights or using weight machines?)

1. Never
2. Rarely
3. Sometimes
4. Frequently
5. Regularly

15) Are you currently pregnant?

1. Yes
2. No
3. Don’t know/prefer not to say

16) What is your height?

1. Height: _____ feet ____ inches OR _____ cm
2. Don’t know/prefer not to say

17) Please estimate your current weight:

1. Weight: _____ lbs OR ______ kg
2. Don’t know/prefer not to say

Vitamin D Survey
[Administered online]

1) Do you **ever** take vitamin D supplements, or a multivitamin containing vitamin D?
   1. No, never
   2. Yes, always
   3. Sometimes/when I remember
   4. I did in the past but I have stopped taking them
   5. Don't know/prefer not to say

**Use automated skip logic (Q2-5 shown if they answered Yes or Sometimes to Q1):*

2) You indicated that you take vitamin D supplements or a multivitamin containing vitamin D. How much vitamin D is in the supplement/multivitamin that you take most frequently? If you are unsure, please check the bottle.
   1. Less than 400 IU
   2. 400-599 IU
   3. 600-999 IU
   4. 1000 IU
   5. Over 1000 IU
   6. Don't know/prefer not to say

3) You indicated that you take vitamin D supplements or a multivitamin containing vitamin D. How often do you take them?
   1. Once a week or less
   2. 2-3 times a week
   3. 4-6 times a week
   4. 1 per day
   5. 2 per day *(e.g. two pills at 400 IU = 800 IU)*
   6. 3 per more a day
   7. Don't know/prefer not to say

4) You indicated that you take vitamin D supplements or a multivitamin containing vitamin D. In which season(s) do you take them?
   1. All year round
   2. In the fall and/or winter months only
   3. In the spring and/or summer months only
   4. Don't know/prefer not to say

5) You indicated that you take vitamin D supplements or a multivitamin containing vitamin D. What motivated you to start taking vitamin D?
   1. A doctor, dietitian or health professional recommended it to me
   2. A friend, family member or colleague recommended it to me
   3. I read about vitamin D (e.g., newspaper, online, magazine, etc.)
   4. I learned about vitamin D in a course or textbook
   5. Someone else in my household takes vitamin D, so I do too

6. It just happens to be in the multivitamin I take
7. Don't know/prefer not to say
8. Other (please specify): ___________

OR
**Use automated skip logic (Q6 shown if they answered No or I used to but stopped to Q1):*

6) You indicated that you **do not** take vitamin D supplements or a multivitamin containing vitamin D. What is your primary reason for not taking vitamin D?
   1. No reason/I never thought about it
   2. I feel that I get enough vitamin D from my diet
   3. I feel that I get enough vitamin D from the sun
   4. I prefer not to take supplements/vitamins
   5. I do not see the point of doing so
   6. Don't know/prefer not to say
   7. Other (please specify): ____________

7) If you are a parent, do you give your child(ren) vitamin D supplements (including drops, pills/tablets, or a multivitamin containing vitamin D)?
   1. No, never
   2. Yes, always
   3. Sometimes/when I remember
   4. I did in the past but I have stopped
   5. Don't know/prefer not to say

**Use automated skip logic (Q8-9 shown if they answered Yes or Sometimes to Q7):*

8) You indicated that you give your child(ren) vitamin D supplements or a multivitamin containing vitamin D. How much vitamin D is in the supplement/multivitamin that you give your child(ren) most frequently? If you are unsure, please check the bottle.
   1. Less than 400 IU
   2. 400-599 IU
   3. 600-999 IU
   4. 1000 IU
   5. Over 1000 IU
   6. Don't know/prefer not to say

9) You indicated that you give your child(ren) vitamin D supplements or a multivitamin containing vitamin D. How often do you give your child vitamin D supplements (including drops, pills/tablets, or a multivitamin containing vitamin D)?
   1. Once a week or less
   2. 2-3 times a week
   3. 4-6 times a week
   4. 1 per day
   5. 2 per day *(e.g. two 400 IU pills = 800 IU)*

6. 3 or more per day
7. Unsure/prefer not to say

**Use automated skip logic (Q10 shown if they answered No or I used to to Q7):*

10) You indicated that you **do not** give your child(ren) vitamin D supplements or a multivitamin containing vitamin D. What is your primary reason for not giving them vitamin D?
    8. No reason/I never thought about it
    9. I feel that they get enough vitamin D from they diet
    10. I feel that they get enough vitamin D from the sun
    11. I prefer not to give my child(ren) supplements/vitamins
    12. I do not see the point of doing so
    13. Don't know/prefer not to say
    14. Other (please specify): ____________

Please indicate how much you agree or disagree with the following statements.

11) From a health perspective, it is important for me to take vitamin D supplements in the spring/summer months in Canada.
    1. Strongly disagree
    2. Disagree somewhat
    3. Neither agree nor disagree
    4. Agree somewhat
    5. Strongly agree

12) From a health perspective, it is important for me to take vitamin D supplements in the fall/winter months in Canada.
    1. Strongly disagree
    2. Disagree somewhat
    3. Neither agree nor disagree
    4. Agree somewhat
    5. Strongly agree

13) From a health perspective, it is important for me to consume milk, dairy and/or fortified dairy alternatives (e.g. enriched soy/almond/coconut/goat's milk) every day.
    1. Strongly disagree
    2. Disagree somewhat
    3. Neither agree nor disagree
    4. Agree somewhat
    5. Strongly agree

14) Consuming milk, dairy and/or fortified dairy alternatives (e.g. enriched soy/almond/coconut/goat's milk) every day is a challenge for me.
    1. Strongly disagree
    2. Disagree somewhat

3. Neither agree nor disagree
4. Agree somewhat
5. Strongly agree

15) From a health perspective, it is important for me to regularly take vitamin D supplements or multivitamins containing vitamin D.
    1. Strongly disagree
    2. Disagree somewhat
    3. Neither agree nor disagree
    4. Agree somewhat
    5. Strongly agree

16) From a health perspective, it is important for me to get some sun exposure every day.
    1. Strongly disagree
    2. Disagree somewhat
    3. Neither agree nor disagree
    4. Agree somewhat
    5. Strongly agree

Now we are going to ask you some general questions regarding your knowledge of vitamin D. Please answer to the best of your ability. We do not expect you to know all of the things we are asking about! If you do not know the answer to a question, please answer honestly and do not look it up before responding. Your responses will help us to understand what young adults do and do not know about vitamin D.

17) According to Health Canada, what is the recommended daily intake of vitamin D for adults of your age group? This is also known as the "Recommended Dietary Allowance" or RDA. **Please enter a number** in one of the boxes below. If you are unsure, please do your best to estimate.
    1. _______ International Units (IU) per day **OR** _____ micrograms (*ug)* per day
    2. Don't know/prefer not to say

18) Which of the following nutrients does your body need **in order to absorb calcium?** *(Please select only one response).*
    1. Vitamin A
    2. Vitamin B_{12}
    3. Vitamin B_6
    4. Vitamin C
    5. Vitamin D
    6. Vitamin E
    7. Vitamin K
    8. Thiamin
    9. Iron
    10. Magnesium

11. Phosphorus
12. Potassium
13. Zinc
14. Don't know/prefer not to say

19) Which of the following are good dietary sources of vitamin D? Please check all that apply.
    1. Fruits
    2. Vegetables
    3. Poultry (e.g. chicken, turkey)
    4. Meat (e.g. beef, pork)
    5. Liver
    6. Oily fish (e.g. salmon, sardines, herring)
    7. Fish oil (e.g. cod liver oil)
    8. Egg yolks
    9. Egg whites
    10. Nuts, seeds
    11. Bread, grain products
    12. Cow's milk
    13. Butter
    14. Margarine
    15. Other dairy products (e.g. cheese, yogurt)
    16. Fortified /enriched non-dairy products (e.g. enriched soy/almond/coconut/goat's milk)
    17. Unenriched non-dairy products (e.g. unenriched soy/almond/coconut/goat's milk)
    18. Fortified juices (e.g. fortified orange juice)
    19. Other, please specify: ________

20) Do you know of any **non-dietary** sources of vitamin D? (Aside from foods, beverages or supplements?)
    1. Yes; please specify:
    2. Don't know/prefer not to say

21) Vitamin D comes in more than one form. Do you know which form comes from the sun and is normally found in vitamin D supplements?
    1. Vitamin D_1
    2. Vitamin D_2
    3. Vitamin D_3
    4. I did not know there were different types of vitamin D
    5. Don't know/prefer not to say

22) According to Health Canada, what is the maximum amount of vitamin D that adults should consume per day from dietary sources (including foods, beverages and supplements)? This is also known as the "Tolerable Upper Limit" or UL. Please **enter a number** in one of the boxes below. If you are unsure, please do your best to estimate.
    1. _____ International Units (IU) OR _____ micrograms (*ug*) per day

2. Don't know/prefer not to say

Now we are going to ask you a few questions about your feelings towards sun exposure and tanning. Please answer honestly.

23) Which of the following best describes how you usually behave when you are spending time in the sun during the spring/summer:
    1. I don't do anything special
    2. I usually wear a hat to keep the sun off my face/head
    3. I usually apply sunscreen
    4. I try to minimize my time in direct sunlight (select a partly/fully shady spot if I can)
    5. I usually cover up as much as I reasonably can (e.g., wear long sleeves)
    6. Other:

24) If you had the opportunity to spend a day sunbathing (i.e., sit out in the sun for the purpose of tanning), how long would you probably spend in the sun?
    ___ Minutes or
    ____ Hours

25) How often do you wear sunscreen?
    1. Never
    2. Rarely
    3. Only when I know I am going to be spending a long time in the sun (e.g. spending a day at the beach, working outside for the day)
    4. Sometimes
    5. Frequently
    6. Regularly

26) How often do you **take measures to avoid the sun** on hot days (e.g. wearing a hat or long sleeves, covering up, sitting in the shade, or staying indoors)?
    1. Never
    2. Rarely
    3. Sometimes
    4. Frequently
    5. Regularly

27) How often do you sunbathe **to get a tan on a hot sunny day**?
    1. Never
    2. Rarely
    3. Sometimes
    4. Frequently
    5. Regularly

28) How often do you **use tanning beds**?
    1. Never

2. Rarely
3. Sometimes
4. Frequently
5. Regularly

29) When you consider sun exposure, **how much do you think about** each of the following?
   a. Damaging skin/ future skin health
   b. Getting a tan
   c. Getting a sun burn
   d. Developing skin cancer or melanoma
   e. Getting adequate sunlight for overall mood and well-being
   f. Getting adequate sunlight for vitamin D production

Response options:
1. Not at all
2. A little
3. Somewhat
4. A fair amount
5. Very much

30) How much time in direct sunlight is needed in order for our bodies to **make vitamin D in the skin?** Please type a number in the box below to indicate a minimum number of minutes that you think is needed. If you don't know, please do your best to estimate.
   1. Minimum # minutes: ___
   2. Don't know/prefer not to say

31) Which of the following factors influence your body's ability to **make vitamin D from the sun**?
   (Check all that apply)
   1. Age
   2. Sex
   3. Ethnicity
   4. Weight
   5. Cloud cover
   6. Clothing
   7. Sunscreen
   8. Amount of shade/sunlight
   9. Skin type/pigmentation
   10. UV index
   11. Season
   12. Geographic location (latitude)
   13. Don't know/prefer not to say
   14. Other: ________

*Now we are going to ask some questions about **how different nutrients affect your health.** Please answer to the best of your ability. Your responses will help us to understand what young adults know about this topic.*

32) Which of the following nutrients affect your **bone health**?
    (Check all that apply):
    1. Vitamin A
    2. Vitamin B_{12}
    3. Vitamin B_6
    4. Vitamin C
    5. Vitamin D
    6. Vitamin E
    7. Vitamin K
    8. Calcium
    9. Iron
    10. Magnesium
    11. Phosphorus
    12. Potassium
    13. Zinc

33) There has been a lot of research regarding vitamin D. As far as you have heard, which of the following health conditions may result from **not consuming enough vitamin D?**
    1. Alzheimer's disease
    2. Anemia
    3. Blood clots
    4. Bone fractures
    5. Cancer
    6. Celiac disease
    7. Constipation
    8. Crohn's disease
    9. Diabetes (type 1 or 2)
    10. Diarrhea
    11. Dizziness
    12. Heart disease (cardiovascular disease)
    13. Measles
    14. Mumps
    15. Multiple sclerosis
    16. Osteoporosis, osteopenia
    17. Rickets, osteomalacia
    18. Rubella
    19. Tooth decay
    20. Weakened immune system (e.g. flu, bacterial or viral infections)
    21. Other

Now we are going to ask a few questions regarding your attitudes towards a list of behaviours. Please indicate your responses on the scale beside each question.

[Behavioural Expectations]

34) To what extent would you be **likely** to do each of the following behaviours, if the opportunity arose?
   a. Take a vitamin D supplement every day
   b. Eat one to two servings of fish per week
   c. Drink at least two cups of cow's milk (or enriched non-dairy alternative such as soy/almond/coconut/goat's milk) per day
   d. Spend 15-30 minutes in the sun without sunscreen, with at least some skin exposed, twice per week

Response options:
1. Not at all likely
2. A little likely
3. Somewhat likely
4. Fairly likely
5. Very likely

[Subjective norms]

35) **How much do people who are important to you think you should do each of the following over the next month?**
   a. Take a vitamin D supplement every day
   b. Eat one to two servings of fish per week
   c. Drink at least two cups of cow's milk (or enriched non-dairy alternative such as soy/almond/coconut/goat's milk) per day
   d. Spend 15-30 minutes in the sun without sunscreen, with at least some skin exposed, twice per week

Response options:
1. Not at all; they wouldn't care if I did this
2. A little
3. Somewhat
4. A fair amount
5. A lot; they would want me to do this

[Descriptive norms]

36) **a) What percentage of people your age do you think do each of the following? Please type a number between 0 and 100%.**
   a. Take a vitamin D supplement every day
   b. Eat one to two servings of fish per week
   c. Drink at least two cups of cow's milk (or enriched non-dairy alternative such as soy/almond/coconut/goat's milk) per day
   d. Spend 15-30 minutes in the sun without sunscreen, with at least some skin exposed, twice per week

36) b) What percentage of people your age that you know do you think do each of the following? Please type a number between 0 and 100%.

a. Take a vitamin D supplement every day
b. Eat one to two servings of fish per week
c. Drink at least two cups of cow's milk (or enriched non-dairy alternative such as soy/almond/coconut/goat's milk) per day
d. Spend 15-30 minutes in the sun without sunscreen, with at least some skin exposed, twice per week

[Perceived behavioural control]

37) **How easy would it be for you to do each of the following over the next month?**

a. Take a vitamin D supplement every day
b. Eat one to two servings of fish per week
c. Drink at least two cups of cow's milk (or enriched non-dairy alternative such as soy/almond/coconut/goat's milk) per day
d. Spend 15-30 minutes in the sun without sunscreen, with at least some skin exposed, twice per week

Response options:

1. Not at all easy
2. A little easy
3. Somewhat easy
4. Fairly easy
5. Very easy to do if I wanted to

[Behavioural Intentions]

38) **On a scale of 1 to 100, where 0=I do not intend to do this at all and 100=I definitely intend to do this…**
How much do you intend to do each of the following over the next month? Please type a number between 0 and 100%.

a. Take a vitamin D supplement every day
b. Eat one to two servings of fish per week
c. Drink at least two cups of cow's milk (or enriched non-dairy alternative such as soy/almond/coconut/goat's milk) per day
d. Spend 15-30 minutes in the sun without sunscreen, with at least some skin exposed, twice per week

Response Scale:
0=Do not intend to do this at all
100=Definitely intend to do this

[Past behaviour]

39) **<u>How often</u> have you done each of the following over the last month?**

a. Taken a vitamin D supplement every day
b. Eaten one to two servings of fish per week
c. Drank at least two cups of cow's milk (or enriched non-dairy alternative such as soy/almond/coconut/goat's milk) per day
d. Spent 15-30 minutes in the sun without sunscreen, with at least some skin exposed, twice per week

Responses:

1. Never
2. Rarely
3. Sometimes
4. Frequently
5. Every day/each time

The following questions concern your images of people. For example, we all have ideas about what typical movie stars are like or what the typical grandmother is like. We might think of the typical movie star as being pretty or rich, and the typical grandmother as sweet and frail. We are not saying that all movie stars or all grandmothers are exactly alike, but rather that many of them share certain characteristics.

[Prototype evaluation]
This one is broken up into 4 questions labelled 40a-d because otherwise there was no way to have them select all that apply.

40) Think about the **type of person** who does each of the following behaviours.
Pick from the following words to describe **YOUR impression of this type person**, including any negative or positive thoughts that go through your mind as you think about this type of person.

a. Takes a vitamin D supplement every day
b. Eats one to two servings of fish per week
c. Drinks at least two cups of cow's milk per day (or non-dairy alternative such as fortified soy/almond/coconut /goat's milk)
d. Spends 15-30 minutes in the sun without sunscreen, with at least face, arms, leg or back exposed, twice per week

Response options:
Select all that apply:

Smart
Foolish
Worrier
Control freak
Conscientious
Healthy

Wasting their time and/or money
Fit
Health conscious
Diet-obsessed

[Prototype similarity]
41) **In general, how similar are you to the type of person your age who does the following?** (*Please think for a moment about the type of person that does each behaviour before indicating your response).*

a. Takes a vitamin D supplement every day
b. Eats one to two servings of fish per week
c. Drinks at least two cups of cow's milk per day (or non-dairy alternative such as soy/almond/coconut/goat's milk fortified with vitamin D)
d. Spends 15-30 minutes in the sun without sunscreen, with at least face, arms, leg or back exposed, twice per week

Responses:
1. Not at all similar to me
2. A little similar to me
3. Somewhat similar to me
4. Fairly similar to me
5. Very similar to me

Scoring of Knowledge Subscale

The knowledge subscale (max. score=9) was scored by summing the following nine items from the Vitamin D Survey, above:

(1) *According to Health Canada, what is the recommended daily intake of vitamin D for adults of your age group? This is also known as the "Recommended Dietary Allowance" or RDA.* (Open-ended; responses of 600 IU or 15 μg were coded as 1= correct; all other responses coded as 0=incorrect).

(2) *Which of the following nutrients does your body need in order to absorb calcium?* (Categorical; selecting vitamin D was coded as 1=correct; all other responses coded as 0=incorrect).

(3) *Which of the following are good dietary sources of vitamin D? Check any that apply.* (Presented with a list of food categories; computed a proportion correct score where 1.0=max. score).

(4) *Do you know of any non-dietary sources of vitamin D (aside from foods, beverages or supplements)?* (Open-ended; responses related to sun/sunlight/UVB/rays/skin synthesis were coded as 1=correct; all other responses coded as 0=incorrect).

(5) *Vitamin D comes in more than one form. Do you know which form comes from the sun and is also found in most vitamin D supplements?* (Categorical; selecting vitamin D_3 was coded as 1=correct; all other responses coded as 0=incorrect).

(6) *According to Health Canada, what is the maximum amount of vitamin D that adults should consume per day from dietary sources (including foods, beverages and supplements)? This is also known as the "Tolerable Upper Limit" or UL.* (Open-ended; responses of 4000 IU or 100 μg were coded as 1=correct; all other responses coded as 0=incorrect).

(7) *How much time in direct sunlight is needed in order for our bodies to make vitamin D in the skin? Please type a number in the box below to indicate the minimum number of minutes you think is needed.* (Open-ended; responses of 10 through 30 minutes were coded as 1=correct; all other responses coded as 0=incorrect).

(8) *Which of the following factors influence our body's ability to make vitamin D from the sun? Check any that apply.* (Presented with a list of factors; computed a proportion correct score where 1.0=max. score).

(9) *Which of the following nutrients affect your bone health? Check any that apply.*

Fitzpatrick Skin Type Questionnaire
(Fitzpatrick, 1988)

(Administered as part of online survey once only)

1) Which best describes your eye colour?
   1. Light blue, light gray or light green
   2. Blue, gray or green
   3. Hazel or light brown
   4. Brown
   5. Black

2) What is your natural hair colour?
   0. Red or light blonde
   1. Blonde
   2. Dark blonde or light brown
   3. Dark brown
   4. Black

3) Which best describes your skin colour (in unexposed areas)?
   0. Ivory white
   1. Fair or pale
   2. Fair to beige, with golden undertone
   3. Olive or light brown
   4. Dark brown or black

4) How many freckles do you have in unexposed areas?
   1. Many
   2. Several
   3. Few
   4. Very few
   5. None

5) What happens if you stay in the sun too long?
   1. Always burns, blisters and peels
   2. Often burns, blisters and peels
   3. Burns moderately
   4. Burns rarely, if at all
   5. Never burns

6) Does your skin tan?
   0. Never
   1. Seldom
   2. Sometimes
   3. Often
   4. Always

7) How deeply do you tan?

0. Not at all or very little
1. Lightly
2. Moderately
3. Deeply
4. My skin is naturally dark

8) Is your face sensitive to the sun?

0. Very sensitive
1. Sensitive
2. Normal/ Sometimes sensitive
3. Resistant
4. Very resistant/ Never have a problem

9) How often do you tan (e.g. in the sun or in a tanning bed)?

0. Never
1. Seldom
2. Sometimes
3. Often
4. Always

10) When did you last expose your body to the sun or a tanning bed?

0. +3 months ago
1. 2−3 months ago
2. 1−2 months ago
3. Weeks ago
4. Days ago

Note: FFQ was administered online using LimeSurvey. The original table format for food items was retained.

UNIVERSITY OF SASKATCHEWAN
COLLEGE OF PHARMACY AND NUTRITION

FOOD FREQUENCY QUESTIONNAIRE V2

Subject Code

0	0	0	0	0	0	0	0	0
1	1	1	1	1	1	1	1	1
2	2	2	2	2	2	2	2	2
3	3	3	3	3	3	3	3	3
4	4	4	4	4	4	4	4	4
5	5	5	5	5	5	5	5	5
6	6	6	6	6	6	6	6	6
7	7	7	7	7	7	7	7	7
8	8	8	8	8	8	8	8	8
9	9	9	9	9	9	9	9	9

Date DDMMYY

0	0	0	0	0	0
1	1	1	1	1	1
2	2	2	2	2	2
3	3	3	3	3	3
4	4	4	4	4	4
5	5	5	5	5	5
6	6	6	6	6	6
7	7	7	7	7	7
8	8	8	8	8	8
9	9	9	9	9	9

Please Use HB Pencil making sure response bubble is filled in completely.

Please list **nutritional supplements** used in past month, using as much detail as you can remember

BRAND NAME OF SUPPLEMENT OR TYPE (e.g Exact calcium 500 mg & vitamin D 400 IU)	AMOUNT TAKEN (e.g. 1 tablet every other day)

1. We want to know how often you eat or drink certain foods **each month.**
2. Think about a **typical month** not just what you ate this week which might be different.
3. **Medium** portion sizes are given to help you determine the ususal size of the food or drink, and to compare to small and large.
4. If you drink or eat much less (approximately half) than the medium portion size described, then check small. If you drink a large glass of milk every day (approximately 1.5 times the size of medium), then check large.
5. Fill out the form similar to this **example**:
   - If you drink a carton of chocolate milk (500 mL) Monday through Friday, then choose M (medium) and show it as 5 - 6 times per week.

TYPE of FOOD or DRINK	Never or less than 1 per month	1 per month	2-3 per month	1 per week	2 per week	3-4 per week	5-6 per week	1 per day	2+ per day	Medium serving	Serving Size S	M	L
Chocolate	○	○	○	○	○	○	○	○	●	1 cup (8 oz or 250 mL)	○	●	○

TYPE of Food or DRINK	Never or less than 1 per month	1 per month	2 - 3 per month	1 per week	2 per week	3-4 per week	5-6 per week	**1 per day**	2+ per day	**Medium Serving**	**Serving Size**<br>**S**	**M**	**L**
Milk: whole, 2%, 1% or skim										1 cup (8 oz or 250 mL)			
Chocolate Milk										1 cup (8 oz or 250 mL)			
Soy Milk Beverage: Fortified										1 cup (8 oz or 250 mL)			
Soy Drink: Plain (not fortified)										1 cup (8 oz or 250 mL)			
Other plant milks (rice, potato, etc)										1 cup (8 oz or 250 mL)			
Milk in coffee or tea										1 tablespoon			
Milk on cereal (if not included above)										1/2 cup			
Milk shake										1 cup (8 oz or 250 mL)			
Milk dessert (ice cream, pudding)										1/2 cup (one scoop, 1 container)			
Yogurt (milk or soy)										1/2 cup (125 g, 1 container			
Soft Cheese										1 tablespoon			
Hard Cheese										1 cube 2" (2 slices)			
White bread, roll, bun, biscuit bagel, naan, tortilla										1 slice, 1 small roll, 1/2 bagel			
Dark bread, roll, bagel										1 slice, 1 small roll, 1/2 bagel			
Taco chips, nacho chips										1 cup (28g)			
Waffle, pancake, French toast										1 piece (4" round)			
Butter (in any foods eaten)										1 pat 1 teaspoon			
Margarine (in any foods eaten)										1 pat 1 teaspoon			
Tofu										1 cube 2"			

TYPE of Food or DRINK	Never or less than 1 per month	1 per month	2 -3 per month	1 per week	2 per week	3-4 per week	5-6 per week	**1 per day**	2+ per day	Medium Serving	Serving Size S	M	L
Macaroni with cheese										1 cup			
Canned Salmon										2 tablespoons or 1 cup of casserole			
Canned Tuna										2 tablespoons or 1 cup of casserole			
Canned Sardines										2 fish (1/2 can)			
Salmon Steak										90 g (3 oz)			
Other fish: white										90 g (3 oz)			
Other fish: oily										90 g (3 oz)			
Cream soups made with milk										1 cup (8 oz or 250 mL)			
Taco or burrito made with cheese										1 regular taco: 1/2 burrito			
Pizza made with cheese										1 slice			
Lentils, beans, peas										1/2 cup cooked			
Eggs: eaten alone or in other foods										1 large egg			
Potatoes: mashed with milk & margarine										1/2 cup (1scoop)			
Orange Juice: **not** fortifed with calcium, vitamin D										1 cup (8 oz or 250 mL)			
Orange Juice: with calcium, vitamin D										1 cup (8 oz or 250 mL)			
Broccoli, kale, greens										1 cup raw or 1/2 cup cooked			
Seafood: e.g shrimp, crab										1 cup meat			
Beef or Pork										90 g (3 oz)			
Bacon or Sausage										2 slices 2 links			

Goal Setting Measures

[Included in Survey 2 for Intervention Participants Only]

As we learned earlier, the Recommended Dietary Allowance (RDA) for adults is 600 IU per day, and many experts recommend 1,000 IU per day. Consider your current intake. Please take a few moments now to set one goal related to your vitamin D intake. Effective goals are **SMART**. That is: **S**pecific, **M**easurable, **A**chievable, **R**ealistic, and **T**ime-related.

For example, if I wanted to consume more vitamin D than I do now, a SMART goal might be: "I will take one 1,000 IU vitamin D supplement every morning when I drink my orange juice with breakfast." In contrast, a poor goal might be, "I will increase my vitamin D consumption", because it is too vague. The SMART goal in this example is:
Specific - states how I will get my vitamin D (through a supplement)
Measurable - states how much I will increase my intake (1,000 IU per day)
Achievable - I know I can achieve this goal, since it involves a simple action rather than a huge lifestyle change (taking one vitamin supplement per day is simple enough to remember)
Realistic - the likelihood of me meeting my goal is increased because I have tied it to a habit I have already formed (drinking orange juice with breakfast)
Time-related - I have stated when and how often I will take the supplement (every morning when I have breakfast).

Your goal can be related to taking a vitamin D supplement (as in the example above), but it doesn't have to. Some other ways you might plan to increase your intake are: Eating one or two servings of fish per week. Think about: What kind of fish do you like? How will you incorporate it into your current dietary habits? (E.g. Will you replace a meat meal with fish once per week? Will you switch your usual lunch with a tuna or salmon salad sandwich twice a week?) Drinking one extra glass of milk (or vitamin D-fortified milk alternative) per day.

Make sure you specify how and when in the day you will do this! (E.g. Will you drink a glass of milk with dinner? Will you start ordering a latte every morning instead of your regular coffee? Will you make a smoothie with milk at breakfast?) Taking a daily multivitamin that contains vitamin D. When will you take your multivitamin? (E.g. Will you take it every morning or every evening?)

How will you remember to take it? TIP: tying your goal behaviour to a related, existing habit will help you remember it. Think about what habits you already have. For instance, if you drink a certain beverage (e.g. coffee, juice) or eat a certain food (e.g. cereal) every morning, you could plan to take your multivitamin or vitamin D supplement at that time. Personally, I find that pouring my orange juice now reminds me to take my vitamin D, since I have formed the habit of using it to swallow my vitamins every morning. Another idea: you could choose to keep your vitamins on your bedside table or beside your toothbrush, so that you remember to take them when you wake up, or when you brush your teeth. Your own idea – be creative and choose something that will work for YOU!

Now it's time to set one SMART goal that would work for you. Please type your goal in the box below. If you feel that you already consume enough vitamin D, please set a SMART goal outlining how you will continue to maintain your current intake.

Goal Setting Check-in Email (Week 9)

We wanted to touch base regarding the goal you set as part of the second online survey. If you remember, we asked you to set a SMART goal to help increase your vitamin D intake. Now that you've been using the Vitamin D Calculator app, we are wondering two things. Please respond to the following quick questions by responding to this email.

1. Did seeing your vitamin D results in the feedback page of the app lead you to increase your commitment to your goal? (Yes/No)

2. Did seeing your vitamin D results lead you to modify or change your goal? (Yes/No)
    - If yes, how so?
    - What is your new goal?

We appreciate you taking the time to answer these questions and to continue to complete your recordings! If you have any questions, don't hesitate to ask by replying to this email.

Goal Setting Questions

1) When you completed the previous online survey, you created a SMART goal to help you increase your intake of vitamin D. Do you remember what your original goal was?
    1. Yes, my original goal was: *[Open-ended]*
    2. I do not remember my goal
    3. I did not set a goal

2) After receiving feedback regarding your vitamin D intake from the app, you may have made changes to your goal, or made a new goal. Did you make changes to your original goal at any point during the study?
    1. Yes
    2. No

3) You indicated that you made changes to your original goal. What was your most recent goal? [Open-ended]

4) Sometimes things happen that make it hard for us to reach our goals, despite our best intentions. How well did you do at meeting your goal? If you made changes to your original vitamin D goal, please answer in relation to your most recent goal.
    1. Not at all – I did not meet any aspects of my goal
    2. A little
    3. Somewhat
    4. Fairly well
    5. Very well – I met my goal completely

Feedback Questions

Finally, we'd like some feedback on this study, including the video, the newsletters we sent you, and your experiences using the Vitamin D Calculator app. Please answer honestly based on your own opinions and experiences.

During the second online survey, you watched a short video about vitamin D by registered dietitian Leslie Beck.

1) How much would you say watching the video influenced you to make changes to your dietary intake of foods containing vitamin D?
   1. Did not influence me at all
   2. Did not influence me very much
   3. Influenced me somewhat
   4. Influenced me very much
   5. I do not remember

2) How much would you say watching the video influenced you to make changes to your dietary intake of foods containing calcium?
   1. Did not influence me at all
   2. Did not influence me very much
   3. Influenced me somewhat
   4. Influenced me very much
   5. I do not remember

3) How much would you say watching the video influenced you to take a supplement or multivitamin containing vitamin D?
   1. Did not influence me at all
   2. Did not influence me very much
   3. Influenced me somewhat
   4. Influenced me very much
   5. I do not remember

4) How much would you say watching the video influenced to spend more time in the sun?
   1. Did not influence me at all
   2. Did not influence me very much
   3. Influenced me somewhat
   4. Influenced me very much
   5. I do not remember

After watching the video, we provided you with some information about vitamin D. This information was presented as a series of screens with some facts about vitamin D. This was included as part of the second online survey.

5) How much would you say reading this information influenced you to make changes to your dietary intake of foods containing vitamin D?

1. Did not influence me at all
2. Did not influence me very much
3. Influenced me somewhat
4. Influenced me very much
5. I do not remember

6) How much would you say using reading this information influenced you to make changes to your dietary intake of foods containing calcium?

1. Did not influence me at all
2. Did not influence me very much
3. Influenced me somewhat
4. Influenced me very much
5. I do not remember

7) How much would you say reading this information influenced you to take a supplement or multivitamin containing vitamin D?

1. Did not influence me at all
2. Did not influence me very much
3. Influenced me somewhat
4. Influenced me very much
5. I do not remember

8) How much would you say reading this information influenced you to spend more time in the sun?

1. Did not influence me at all
2. Did not influence me very much
3. Influenced me somewhat
4. Influenced me very much
5. I do not remember

9) Did you read the "Vitamin D tricks and tips" newsletters that we sent you by email attachment?

1. Yes, I read all 3 newsletters
2. I read one or two of the 3 newsletters
3. No, I did no read the newsletters

10) How much would you say receiving the “Vitamin D Tips & Tricks” newsletters influenced you to make changes to your dietary intake of foods containing vitamin D?

1. Did not influence me at all
2. Did not influence me very much
3. Influenced me somewhat

4. **4.** Influenced me very much
5. **5.** N/A – I did not read the newsletters

11) How much would you say receiving the "Vitamin D Tips & Tricks" newsletters influenced you to make changes to your dietary intake of foods containing calcium?

1. Did not influence me at all
2. Did not influence me very much
3. Influenced me somewhat
4. Influenced me very much
5. N/A – I did not read the newsletters

12) How much would you say receiving the "Vitamin D Tips & Tricks" newsletters influenced you to take a supplement or multivitamin containing vitamin D?

1. Did not influence me at all
2. Did not influence me very much
3. Influenced me somewhat
4. Influenced me very much
5. N/A – I did not read the newsletters

13) How much would you say receiving the "Vitamin D Tips & Tricks" newsletters influenced you to spend more time in the sun?

1. Did not influence me at all
2. Did not influence me very much
3. Influenced me somewhat
4. Influenced me very much
5. N/A – I did not read the newsletters

Now we'll ask you a few questions regarding the Vitamin D Calculator app. How much did you like using the app?

14) How much did you **like** using the app?

1. Did not like using it at all
2. Did not like using it very much
3. Liked using it somewhat
4. Liked using it very much
5. N/A – I did not use the app

15) How **easy** was it to use the app?

1. Very difficult
2. Somewhat difficult
3. Somewhat easy
4. Very easy
5. N/A – I did not use the app

16) How much would you say using the app **influenced** you to make changes to your dietary intake of foods containing vitamin D?

1. Did not influence me at all
2. Did not influence me very much
3. Influenced me somewhat
4. Influenced me very much
5. N/A – I did not use the app

17) How much would you say using the app **influenced** you to make changes to your dietary intake of foods containing calcium?

1. Did not influence me at all
2. Did not influence me very much
3. Influenced me somewhat
4. Influenced me very much
5. N/A – I did not use the app

18) How much would you say the app influenced you to take a supplement or multivitamin containing vitamin D?

1. Did not influence me at all
2. Did not influence me very much
3. Influenced me somewhat
4. Influenced me very much
5. N/A – I did not use the app

19) How much would you say the app influenced you to spend more time in the sun?

1. Did not influence me at all
2. Did not influence me very much
3. Influenced me somewhat
4. Influenced me very much
5. N/A – I did not use the app

20) What is one thing you <u>liked</u> about using the vitamin D calculator app? *[Open-ended]*

21) What is one thing we could do to <u>improve</u> the vitamin D calculator app? *[Open-ended]*

22) Do you have any other **comments or feedback** regarding your experience using the Vitamin D Calculator app? *[Open-ended]*
