# Supplementary material for: A randomized, controlled trial evaluating the efficacy of an online intervention targeting vitamin D intake, knowledge and status among young adults
Source: Int J Behav Nutr Phys Act. 2016 Nov 11;13:116. doi: 10.1186/s12966-016-0443-1 (PMC5106840; doi:10.1186/s12966-016-0443-1)
Supplement: Additional file 3: — Additional Tables. Intervention study supplementary tables A.1, A.2 and A.3. (PDF 138 kb) [file 12966_2016_443_MOESM3_ESM.pdf]

Additional File 2: Additional Tables

Table A.1: Means and Standard Deviations for Perceived Importance of Vitamin D-Related Behaviours among Intervention Study Participants Over Time (n=90)

Variable*	Intervention (n=41) M (SD)	Control (n=49) M (SD)
**From a health perspective, it is important for me to regularly take supplements or multivitamins containing vitamin D.**		
Time 1 (Baseline)	3.17 (1.09)	2.98 (1.25)
Time 2	3.56 (1.14)	2.98 (1.23)
Time 3 (Follow-up)	3.71 (1.12)	3.24 (1.28)
**From a health perspective, it is important for me to take vitamin D supplements in the spring/summer months in Canada.**		
Time 1 (Baseline)	2.88 (1.05)	2.53 (1.08)
Time 2	2.98 (1.17)	2.67 (1.09)
Time 3 (Follow-up)	2.95 (1.07)	2.82 (1.32)
**From a health perspective, it is important for me to take vitamin D supplements in the fall/winter months in Canada.**		
Time 1 (Baseline)	4.07 (0.88)	3.67 (1.09)
Time 2	4.27 (0.95)	3.73 (1.10)

Time 3 (Follow-up)	4.12 (1.01)	3.78 (1.25)
**From a health perspective, it is important for me to consume milk, dairy and/or fortified non-dairy alternatives every day.**		
Time 1 (Baseline)	4.17 (1.14)	3.92 (1.22)
Time 2	4.12 (0.93)	4.12 (0.95)
Time 3 (Follow-up)	4.12 (0.98)	4.29 (0.84)
**Consuming milk, dairy, and/or fortified non-dairy alternatives is a challenge for me.**		
Time 1 (Baseline)	2.44 (1.48)	2.49 (1.45)
Time 2	3.17 (1.47)	2.33 (1.18)
Time 3 (Follow-up)	3.22 (1.26)	2.94 (1.38)
**From a health perspective, it is important for me to get some sun exposure every day.**		
Time 1 (Baseline)	4.05 (1.05)	4.14 (0.94)
Time 2	4.07 (0.99)	4.12 (0.86)
Time 3 (Follow-up)	3.95 (112)	4.43 (0.76)

*Response range 1-5 (*1=strongly disagree; 5=strongly agree*)

Table A.2: Response Frequencies for Feedback Survey among Intervention Group (n=41)

Variable	Frequency % (n)
**How much would you say watching the video influenced you to make changes to your dietary intake of foods containing vitamin D?**	
Did not influence me at all	17.1% (7)
Did not influence me very much	31.7% (13)
Influenced me somewhat	36.6% (15)
Influenced me very much	12.2% (5)
I do not remember	2.4% (2)
**How much would you say watching the video influenced you to make changes to your dietary intake of foods containing calcium?**	
Did not influence me at all	19.5% (8)
Did not influence me very much	29.3% (12)
Influenced me somewhat	41.5% (17)
Influenced me very much	7.3% (3)
I do not remember	2.4% (1)
**How much would you say watching the video influenced you to take a supplement or multivitamin containing vitamin D?**	
Did not influence me at all	31.7% (13)
Did not influence me very much	24.4% (10)

Influenced me somewhat	29.3% (12)
Influenced me very much	12.2% (5)
I do not remember	2.4% (1)
**How much would you say watching the video influenced to spend more time in the sun?**	
Did not influence me at all	22.0% (9)
Did not influence me very much	26.8% (11)
Influenced me somewhat	34.1% (14)
Influenced me very much	14.6% (6)
I do not remember	2.4% (1)
After watching the video, we provided you with some information about vitamin D. **How much would you say reading this information influenced you to make changes to your dietary intake of foods containing vitamin D?**	
Did not influence me at all	14.6% (6)
Did not influence me very much	24.4% (10)
Influenced me somewhat	29.3% (12)
Influenced me very much	24.4% (10)
I do not remember	7.3% (3)
**How much would you say using reading this information influenced you to make changes to your dietary intake of foods containing calcium?**	
Did not influence me at all	12.2% (5)
Did not influence me very much	34.1% (14)

Influenced me somewhat	31.7% (13)
Influenced me very much	14.6% (6)
I do not remember	7.3% (3)
**How much would you say reading this information influenced you to take a supplement or multivitamin containing vitamin D?**	
Did not influence me at all	24.4% (10)
Did not influence me very much	24.4% (10)
Influenced me somewhat	26.8% (11)
Influenced me very much	17.1% (7)
I do not remember	7.3% (3)
**How much would you say reading this information influenced you to spend more time in the sun?**	
Did not influence me at all	19.5% (8)
Did not influence me very much	24.4% (10)
Influenced me somewhat	34.1% (14)
Influenced me very much	14.6% (6)
I do not remember	7.3% (3)
**How much would you say receiving the "Vitamin D Tips & Tricks" newsletters influenced you to make changes to your dietary intake of foods containing vitamin D?**	
Did not influence me at all	2.4% (1)
Did not influence me very much	29.3% (12)

Influenced me somewhat	19.5% (8)
Influenced me very much	19.5% (8)
N/A – I did not read the newsletters	22.0% (9)
I do not remember	7.3% (3)

How much would you say receiving the "Vitamin D Tips & Tricks" newsletters influenced you to make changes to your dietary intake of foods containing calcium?

Did not influence me at all	4.9% (2)
Did not influence me very much	26.8% (11)
Influenced me somewhat	29.3% (12)
Influenced me very much	9.8% (4)
N/A – I did not read the newsletters	22.0% (9)
I do not remember	7.3% (3)

How much would you say receiving the "Vitamin D Tips & Tricks" newsletters influenced you to take a supplement or multivitamin containing vitamin D?

Did not influence me at all	12.2% (5)
Did not influence me very much	19.5% (8)
Influenced me somewhat	22.0% (9)
Influenced me very much	17.1% (7)
N/A – I did not read the newsletters	22.0% (9)
I do not remember	7.3% (3)

How much would you say receiving the "Vitamin D Tips & Tricks" newsletters influenced you to spend more time in the sun?

Did not influence me at all	14.6% (6)
Did not influence me very much	17.1% (7)
Influenced me somewhat	24.4% (10)
Influenced me very much	19.5% (8)
N/A – I did not read the newsletters	22.0% (9)
I do not remember	7.3% (3)

How much would you say using the app influenced you to make changes to your dietary intake of foods containing vitamin D?

Did not influence me at all	24.4% (10)
Did not influence me very much	14.6% (6)
Influenced me somewhat	26.8% (11)
Influenced me very much	24.4% (10)
N/A – I did not use the app	9.8% (4)

How much would you say using the app influenced you to make changes to your dietary intake of foods containing calcium?

Did not influence me at all	29.3% (12)
Did not influence me very much	17.1% (7)
Influenced me somewhat	24.4% (10)
Influenced me very much	19.5% (8)

N/A – I did not use the app	9.8% (4)
**How much would you say the app influenced you to take a supplement or multivitamin containing vitamin D?**	
Did not influence me at all	43.9% (18)
Did not influence me very much	12.2% (5)
Influenced me somewhat	17.1% (7)
Influenced me very much	17.1% (7)
N/A – I did not use the app	9.8% (4)
**How much would you say the app influenced you to spend more time in the sun?**	
Did not influence me at all	43.9% (18)
Did not influence me very much	14.6% (6)
Influenced me somewhat	24.4% (10)
Influenced me very much	7.3% (3)
N/A – I did not use the app	9.8% (4)
**How much did you like using the app?**	
Did not like using it at all	19.5% (8)
Did not like using it very much	19.5% (8)
Liked using it somewhat	39.0% (16)
Liked using it very much	7.3% (3)

N/A – I did not use the app	14.6% (6)
**How easy was it to use the app?**	
Very difficult	9.8% (4)
Somewhat difficult	26.8% (11)
Somewhat easy	22.0% (9)
Very easy	31.7% (13)
N/A – I did not use the app	9.8% (4)

Table A.3: Mean Rankings of Intervention Components on Perceived Influence of Behaviour Change (Feedback Survey, n=41)

**Dependent Variable[*]**	**Intervention Components**			
How much did the [intervention component] influence you to…	**Intervention video M (SD)**	**Information slides M (SD)**	**Tips & Tricks newsletters M (SD)**	**Vitamin D Calculator app M (SD)**
**Make changes to dietary intake of foods containing vitamin D** (n=25)	2.48 (0.82)	2.80 (1.00)	2.80 (0.91)	2.76 (1.05)
**Make changes to dietary intake of foods containing calcium** (n=24)	2.50 (0.83)	2.67 (0.82)	2.67 (0.82)	2.58 (1.10)
**Take a supplement or multivitamin containing vitamin** D (n=25)	2.36 (1.00)	2.52 (1.05)	2.60 (1.04)	2.36 (1.22)
**Spend more time in the sun** (n=26)	2.65 (0.98)	2.58 (1.03)	**2.69 (1.05)[a]**	**2.08 (1.09)[a]**

[a] Significantly different at $p<0.008$

[*] Response range 1-4 *(1=did not influence me at all; 4=influenced me very much)*
